# The Amplitude of Diaphragm Compound Muscle Action Potential Correlates With Diaphragmatic Excursion on Ultrasound and Pulmonary Function After Supraclavicular Brachial Plexus Block

**DOI:** 10.3389/fmed.2021.744670

**Published:** 2022-03-21

**Authors:** Xiuxia Bao, Tao Liu, Haorong Feng, Yeke Zhu, Yingying Wu, Xianghe Wang, Xianhui Kang

**Affiliations:** ^1^Department of Anesthesiology, The First Affiliated Hospital, Zhejiang University School of Medicine, Hangzhou, China; ^2^Department of Anesthesiology, The First People' Hospital of Huzhou, Huzhou, China; ^3^Department of Anesthesiology, South Taihu Hospital of Huzhou, Huzhou, China; ^4^Department of Anesthesiology, Huzhou Fourth Hospital, Huzhou, China; ^5^Department of Anesthesiology, The 98th Clinical College of People's Liberation Army (PLM), Anhui Medical University, Huzhou, China

**Keywords:** diaphragm compound muscle action potential (CMAP), supraclavicular brachial plexus block, phrenic nerve conduction studies, hemidiaphragmatic excursion, pulmonary function

## Abstract

**Objective:**

This prospective, double-blind, randomized study assessed (1) the associations between diaphragm compound muscle action potential (CMAP), hemidiaphragmatic excursion, and pulmonary function after supraclavicular brachial plexus block (SCBPB) and (2) diagnostic efficacy of pulmonary function for hemidiaphragmatic paralysis evidenced by diaphragm CMAP as an assessment of diaphragm strength was evaluated.

**Methods:**

Eighty-six patients were scheduled for the removal of hardware after healing of a right upper limb fracture distal to the shoulder who were randomly assigned in a 1:1 ratio to two groups: Group A (diaphragmatic excursion), or Group B (pulmonary function). Phrenic nerve conduction studies (PNCSs), M-mode ultrasonography of the diaphragm, and pulmonary function tests (PFTs) were performed before and 30 min after SCBPB. PNCSs were used to determine the latency and amplitude of diaphragm CMAP. Ultrasonography of the diaphragm was performed with patients in a supine position using a low-frequency probe over the subcostal space at the midclavicular line. The diaphragmatic excursion was measured during quiet breathing and deep breathing. Pulmonary function, i.e., forced vital capacity (FVC), predicted value of FVC, and forced expiratory flow in the first second (FEV1), was measured with spirometry. Receiver Operating Characteristic (ROC) curve analysis was used to assess the diagnostic efficacy of pulmonary function for hemidiaphragmatic paralysis evidenced by diaphragm CMAP as an assessment of diaphragm strength.

**Results:**

There were significant associations between the reduction in amplitude of diaphragm CMAP and reductions in diaphragmatic excursion during quiet breathing (*r* = 0.70, *p* < 0.01) and deep breathing (*r* = 0.63, *p* < 0.01) when expressed as a percentage of baseline values. There were significant associations between the reduction in amplitude of diaphragm CMAP and reductions in FVC (*r* = 0.67, *p* < 0.01), FVC% (*r* = 0.67, *p* < 0.01), and FEV1 (*r* = 0.62, *p* < 0.01), when expressed as percentage of baseline values. The area under the ROC curve for FVC was 0.86. A decrease of >8.4% in FVC compared to pre-block predicted hemidiaphragmatic paralysis (determined by diaphragm CMAP) with sensitivity and specificity of 79.2 and 100%, respectively.

**Conclusions:**

The relative reduction in diaphragm CMAP amplitude after SCBPB was correlated with relative reductions in diaphragmatic excursion and pulmonary function. FVC has potential as a useful diagnostic indicator of hemidiaphragmatic paralysis, evidenced by diaphragm CMAP, after SCBPB. These data establish diaphragm CMAP as a direct and objective index of diaphragmatic paralysis after SCBPB.

## Introduction

Brachial plexus block is an effective method for providing anesthesia and post-operative analgesia in patients who underwent surgery of the upper limb. Compared to general anesthesia, brachial plexus block is associated with reduced opioid consumption and opioid-related side effects, decreased length of stay in the post-anesthesia care unit (PACU), and improved patient satisfaction (1). However, brachial plexus block may result in ipsilateral phrenic nerve block and hemidiaphragmatic paresis (2). The incidence of ipsilateral hemidiaphragmatic palsy in the interscalene brachial plexus block is almost 100% (3). However, the distance between the phrenic nerve and the brachial plexus increases caudally to the cricoid cartilage; therefore, the incidence of ipsilateral hemidiaphragmatic palsy in supraclavicular brachial plexus block (SCBPB) is 36–67% (2).

The diaphragm is a main inspiratory muscle, accounting for 75% of the total tidal volume in quiet breathing (4). Healthy individuals at rest are able to tolerate hemidiaphragmatic paresis, but they may experience exercise-associated dyspnea and reduced exercise tolerance (5–8). Patients with unilateral paralysis of the diaphragm usually have no symptoms, as the contralateral diaphragm can compensate. In some patients, clinical signs of respiratory failure after diaphragmatic paralysis cannot be resolved through compensation by the contralateral diaphragm. In one report, a patient with no obesity or history of respiratory disease experienced dyspnea in the post-operative recovery unit after total shoulder replacement (9). In patients with obesity and respiratory diseases, dyspnea and decreased blood oxygen saturation may be more serious.

Diagnosis of diaphragmatic paralysis is essential to avoid respiratory morbidity and can be achieved with a variety of tests. Spirometry techniques are commonly used to measure pulmonary function by assessing forced vital capacity (FVC), predicted value of FVC, and forced expiratory flow in the first second (FEV1). The quality of the spirometry test is influenced by the effort and cooperation of a patient. With the evolution of ultrasound technology, M-mode imaging has been used to identify abnormal diaphragm movement/position during quiet or deep breathing (4); however, ultrasound does not account for the influence of the contralateral diaphragm and accessory respiratory muscles. Phrenic nerve conduction can overcome these challenges. Phrenic nerve conduction is an emerging technique that can be used to evaluate diaphragmatic dysfunction (10, 11) by measuring the change of diaphragm compound muscle action potentials (CMAPs).

Physicians should be aware of diaphragmatic paralysis caused by brachial plexus block, diagnosis of diaphragmatic paralysis, and that early detection of diaphragmatic paralysis can prevent the onset of dyspnea (9). The objective of this prospective, double-blind, randomized study was to assess the associations between diaphragm CMAP, hemidiaphragmatic excursion, and pulmonary function after SCBPB. The diagnostic efficacy of pulmonary function for hemidiaphragmatic paralysis evidenced by diaphragm CMAP as an assessment of diaphragm strength was evaluated.

## Methods

### Patients

Patients scheduled for the removal of hardware after healing of right upper limb fracture distal to the shoulder between September 1, 2019 and January 30, 2020 at the 98th Hospital of the People's Liberation Army (PLA) were eligible for this study. Inclusion criteria were as follows: (1) aged 18–60 years; (2) American Society of Anesthesiologists physical status class I or II; (3) body weight 45–85 kg; and (4) consented to SCBPB. Exclusion criteria were as follows: (1) history of the cardiopulmonary disease; (2) body mass index (BMI) >30 kg/m^2^; (3) sensory or motor impairment; (4) coagulopathy or severe renal or hepatic failure; (4) known allergy to local anesthetics; (5) extremely atypical diaphragm CMAP; or (6) mild block.

Included patients were randomly assigned in a 1:1 ratio (using a computer-generated random number table and the sealed envelope system for allocation concealment) to two groups: Group A, in which diaphragm CMAP and diaphragmatic excursion were measured, or Group B, in which diaphragm CMAP and pulmonary function were measured. Two groups of patients were required as it takes 2 min to measure diaphragmatic excursions and 3 min to measure pulmonary function. We could not adjust for the differences in the time it takes to obtain these measurements; therefore, both measurements could not be obtained from a single group of patients.

This prospective, double-blind, randomized study was approved by the Medical Ethics Committee of the 98th Hospital of the PLA. Written informed consent was obtained from all patients before enrollment. This study was an extension of a clinical trial registered on the Chinese Clinical Registry (http://www.chictr.org.cn/showproj.aspx?proj=16992; ChiCTR-IND-17012166, 2017/7/27).

### Supraclavicular Brachial Plexus Block

Patients were routinely prepared for surgery. Intravenous access was established prior to the brachial plexus block. ECG, oximetry (SPO_2_), and blood pressure (non-invasive) monitoring were performed while patients breathed room air. The patients, the anesthesiologist who managed the patients in the surgery preparation room, and the researchers who collected and analyzed data were blinded to the group allocation.

All the patients were placed in the supine position with the head rotated in the contralateral direction. After sterile skin preparation with 2% chiorhexidine, an ultrasound probe was placed in the supraclavicular fossa to view the brachial plexus and subclavian artery. The skin and subcutaneous tissues were infiltrated with 1 ml of 2% lidocaine. Using the in-plane approach, 0.375% ropivacaine was injected in a lateral-to-medial direction with a sterile 22-gauge, 50-mm insulated nerve stimulating needle. Initially, 15 ml of 0.375% ropivacaine was injected into the “corner pocket” at the junction of the subclavian artery and first rib or pleura (12). The remaining 15 ml of 0.375% ropivacaine was injected into the most superficial portion of the lateral aspect of the cluster formed by the brachial plexus trunks and divisions (13). Adverse events associated with performing the block, such as vascular puncture and dyspnea, were recorded.

The success of the block was assessed after 30 min by an anesthetist who was blinded to the group allocation. The anesthetic effect was categorized as excellent, moderate, or mild. Excellent was defined as no need for supplemental local anesthetics and/or opioids or conversion to general anesthesia. Moderate was defined as the patient can undergo surgery with the need for 1–2 μg/kg fentanyl (14). Mild was defined as the patient was unable to tolerate the pain caused by the skin incision at the initiation of surgery or fentanyl administration did not increase the pain. Patients were followed up for 24 h after completion of infiltration of local anesthetic. Adverse events, such as local anesthetic toxicity, lack of return of normal motor and sensory function, SpO_2_ decline (accounting for a 5% decrease as the patient was breathing room air), difficulty in breathing, and hoarseness, were recorded.

### Diaphragm CMAP With Phrenic Nerve Conduction

Phrenic nerve conduction studies (PNCSs) were performed by a single technician using Cascade software (Cadwell Laboratories, Kennewick, WA, USA) according to previously published protocols (15–17). The patient was placed in the supine position with the head slightly elevated and rotated 30° in the contralateral direction. A bipolar surface stimulating electrode was placed at the posterior border of the right sternomastoid muscle at the level of the cricoid cartilage. One needle was inserted into the diaphragm at the xiphoid process, 16 cm from a second needle that was placed at the ipsilateral costal margin. A ground disc electrode was placed between the stimulator and the recording electrode. The right phrenic nerve was stimulated at end-expiration using rectangular pulses of 0.2 ms duration. The intensity of the stimulus was increased until reproducible response and maximal diaphragm CMAP amplitude were obtained (maximal stimulation). The phrenic nerve was supramaximally stimulated to 10–20% above maximal stimulation. The latency of the diaphragm CMAP, expressed as phrenic nerve conduction time (PNCT), was measured from stimulus to CMAP onset in milliseconds (ms). The amplitude of the diaphragm CMAP was measured from the negative peak to the positive peak of the waveform in microvolts (μV). Mean latency and amplitude of the diaphragm CMAP measured on three tests before and 30 min after SCBPB were used in the analyses. Paralysis of the diaphragm was defined as >50% reduction in the amplitude of the diaphragm CMAP.

Diaphragm CMAP with phrenic nerve conduction was performed after an ultrasound of the diaphragm and spirometry.

### Ultrasound of the Diaphragm

Measurements were performed with the patient in the supine position with the head of the bed at a 30° angle (18) using a SonoSite M-Turbo system with a 2–5 MHz convex transducer. The right hemidiaphragm was visualized by placing the probe over the right anterior subcostal region between the midclavicular and anterior axillary lines. The probe was directed cranially at 30° to the coronal plane. Initially, the right hemidiaphragm was visualized in 2-dimensional (2D) mode with the liver serving as an acoustic window to the right. Subsequently, the probe was raised to the apex of the diaphragmatic dome, and M-mode was used to measure the amplitude of the diaphragmatic excursion during quiet and deep breathing. The means of 3 consecutive measurements before and 30 min after SCBPB were used in the analyses. “Complete paralysis” was defined as >75% reduction in diaphragmatic movement. “Partial paralysis” was defined as a 25–75% reduction in diaphragmatic movement, and “no paralysis” was defined as a <25% reduction in diaphragmatic movement. The number of patients with “paralysis” (partial or complete paralysis) or “no paralysis” was recorded.

### Spirometry

Spirometry (Multi-Functional Spirometer H1-701, Japan) was performed in the surgery preparation room according to the recommendations of the American Thoracic Society (19, 20). Patients were placed in the seated position with the head of the bed at a 90° angle and instructed how to perform the test. The FVC, predicted value for FVC, and FEV1 were measured three times at 2-min intervals before and 30 min after SCBPB. The means of 3 acceptable measurements before and 30 min after SCBPB were used in the analyses.

### Statistical Analysis

Statistical analyses were performed using IBM SPSS Statistics, version 22. Variables are reported as mean ± SD or medians and interquartile range (IQR). Between-group comparisons of baseline demographic and clinical characteristics were conducted using the Student's *t*-test. Associations between diaphragm CMAP amplitude, diaphragmatic excursion, and pulmonary function were evaluated using Pearson correlation analysis. The diagnostic efficacy of pulmonary function for hemidiaphragmatic paralysis determined by diaphragm CMAP was evaluated with Receiver Operating Curve (ROC) analysis. *p* < 0.05 was considered statistically significant.

The sample size required to obtain minimum correlation coefficients between reduction in amplitude of diaphragm CMAP and diaphragmatic excursion during quiet breathing and deep breathing of 0.75 and 0.53, respectively, in Group A and minimum correlation coefficients between reduction in amplitude of diaphragm CMAP and FVC, FVC%, and FEV1 of 0.6, 0.6, and 0.7, respectively, in Group B was calculated using the following formula ($n=4\times {\left[\left(u_{\alpha }+u_{\beta }\right)/\ln\;\left(\frac{1+r}{1-r}\right)\right]}^{2}+3$, *u*_α_ = 1.96, *u*_β_ = 1.282). In total, 34 patients were required in each group. Assuming that 20% of study participants were lost to follow-up or dropped out, 86 patients were enrolled in the study.

The sample size for ROC curve analysis was determined using Power Analysis Software (PASS 11). Based on a preliminary study with 10 patients in each group, to obtain an area under the ROC curve of 0.75 with a statistical power of 80%, an alpha error of 0.05, and assuming that 20% of study participants were lost to follow-up or dropped out, 32 patients were required in Group B.

## Results

### Patient Population

**Figure 1:**
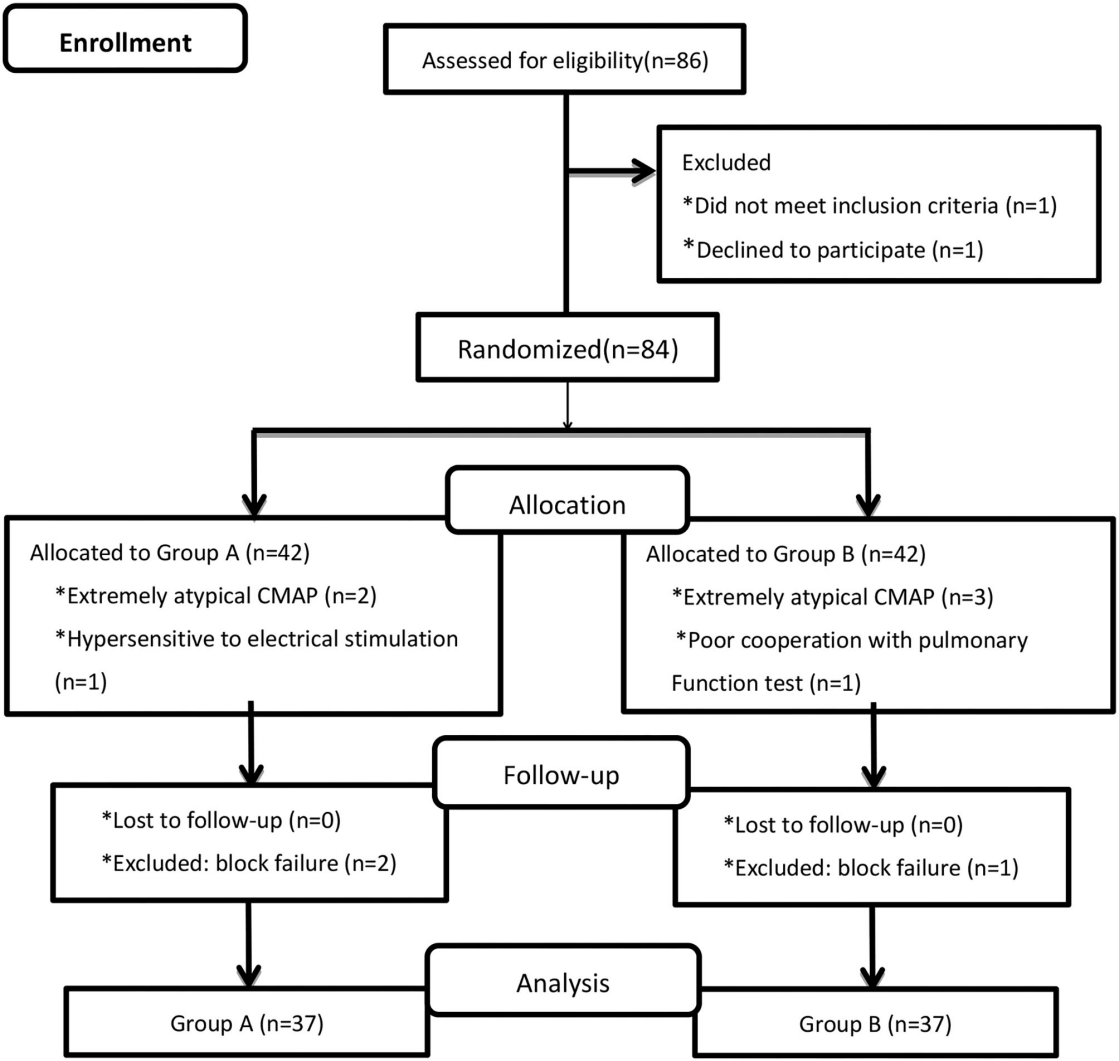
Flowchart of patient selection. CMAP, compound muscle action potential.

A total of 86 patients were randomized, and data for 74 patients were included in the analyses (*n* = 37 in each group; Figure 1). The demographic and clinical characteristics of the patients in Groups A and B were similar (Table 1).

**Table 1:** Baseline demographic and clinical characteristics of the study population.

	**All patients** **(*N* = 74)**	**Group A (*n* = 37)**	**Group B (*n* = 37)**	***P*-value** **(A vs. B)**
Age (years)	41.95 ± 11.32	42.73 ± 11.86	41.16 ± 10.85	*P* = 0.81
Weight (kg)	62.81 ± 11.68	61.19 ± 11.41	64.43 ± 11.87	*P* = 0.78
Height (cm)	166.55 ± 7.66	165.57 ± 7.86	167.54 ± 7.43	*P* = 0.31
BMI(kg/m^2^)	22.52 ± 3.13	22.22 ± 3.15	22.82 ± 3.12	*P* = 0.68
Sex (F/M)	17/57	10/27	7/30	*P* = 0.41
ASA physical status (I/II)	25/49	11/26	14/23	*P* = 0.46
Amplitude (μV)	1034.91 ± 497.96	1039.19 ± 511.06	1030.64 ± 491.52	*P* = 0.61
Amplitude (μV) of diaphragm CMAP after block	382.25 ± 303.77	385.10 ± 334.45	379.40 ± 274.31	*P* = 0.48
Latency (ms)	5.78 ± 1.65	5.69 ± 1.42	5.87 ± 1.86	*P* = 0.15
Quiet excursion (cm)		1.53 ± 0.29 (*P* = 0.16)		
Deep excursion (cm)		7.05 ± 1.03 (*P* = 0.11)		
FVC			3.44 ± 0.75 (*P* = 0.2)	
FVC%			84.48 ± 9.37 (*P* = 0.2)	
FEV1			2.98 ± 0.65 (*P* = 0.17)	

*Values are mean ± SD or number. Group A, diaphragm excursion; Group B, pulmonary function; ASA, American Society of Anesthesiologists; FVC, forced vital capacity; FVC%, predicted value of FVC; FEV1, forced expiratory flow in the first second*.

Overall, 49 of the 74 (66.2%) patients experienced hemidiaphragmatic paralysis evidenced by diaphragm CMAP as the reference standard. Two patients in Group A and one patient in Group B were converted to general anesthesia because the anesthetic effect of the block was mild. The latency and amplitude of the diaphragm CMAP were extremely atypical in two patients in Group A and three patients in Group B, possibly due to stimulation of the brachial plexus. Among these, one patient in Group A was hypersensitive to the electrical stimulation as the amplitude of the diaphragm CMAP was >3,000 μV. This may be because the long thoracic, lateral pectorals, and/or medial pectoral nerves, which are derived from the brachial plexus, were stimulated.

### Associations Between Diaphragm CMAP, Hemidiaphragmatic Excursion, and Pulmonary Function After SCBPB

Respiratory function, diaphragmatic excursion, and diaphragm CMAP are presented in Table 1. There were no differences in the effectiveness of SCBPB between Groups A and B. In Group A (−73.1%) and Group B (−74.6%), the amplitude of diaphragm CMAP was decreased 30 min after SCBPB compared to baseline. In Group A, hemidiaphragmatic excursions during quiet breathing (−40.0%) and deep breathing (−43.4%) were decreased 30 min after SCBPB compared to baseline. In Group B, FVC (−11.9%), FVC% (−11.9%), and FEV1 (−13.2%) were decreased 30 min after SCBPB compared to baseline (Table 2).

**Table 2:** Diaphragm compound muscle action potential, diaphragm excursion during SCBPB, and respiratory function post-SCBPB.

	**All patients** **(*N* = 74)**	**Group A**	**Group B**	***P-*value**
ΔCMAP pre-block to post-block	−73.6% (−86.1~−30.4%)	−73.1% (−87.1~−38.3%)	−74.6% (−84.5~−27.2%)	0.95
ΔPNCT pre-block to post-block	25.1% (4.1~67.6%)	24.2% (4.0~60.6%)	27.7% (4.6~70.3%)	0.67
Δquiet excursion pre-block to post-block		−40.0% (−100~−6.5%)		
Δdeep excursion pre-block to post-block		−43.4% (−57.7~−6.8%)		
ΔFVC pre-block to post-block			−11.9% (−31.2~−4.2%)	
ΔFVC% pre-block to post-block			−11.9% (−31.2~−4.3%)	
ΔFEV_1_ pre-block to post-block			−13.2% (−30.7~−5.1%)	

*SCBPB, supraclavicular brachial plexus block; ΔCMAP, percent reduction in diaphragm compound muscle action potential pre-block to post-block; ΔPNCT, percent reduction in phrenic nerve conduction time pre-block to post-block; Δquiet excursion, percent reduction in quiet breath excursion pre-block to post-block; Δdeep excursion, percent reduction in deep breath excursion pre-block to post-block; ΔFVC, percent reduction in forced vital capacity pre-block to post-block; ΔFVC%, percent reduction in predicted FVC pre-block to post-block; ΔFEV1, the percent reduction in forced expiratory flow in the first second*.

Five patients (13.5%) in Group A vs. three patients (8.1%) in Group B reported transient dyspnea and oxygen desaturation 10 min after SCBPB. Symptoms were significantly improved after oxygen inhalation through a nasal catheter.

**Figure 2:**
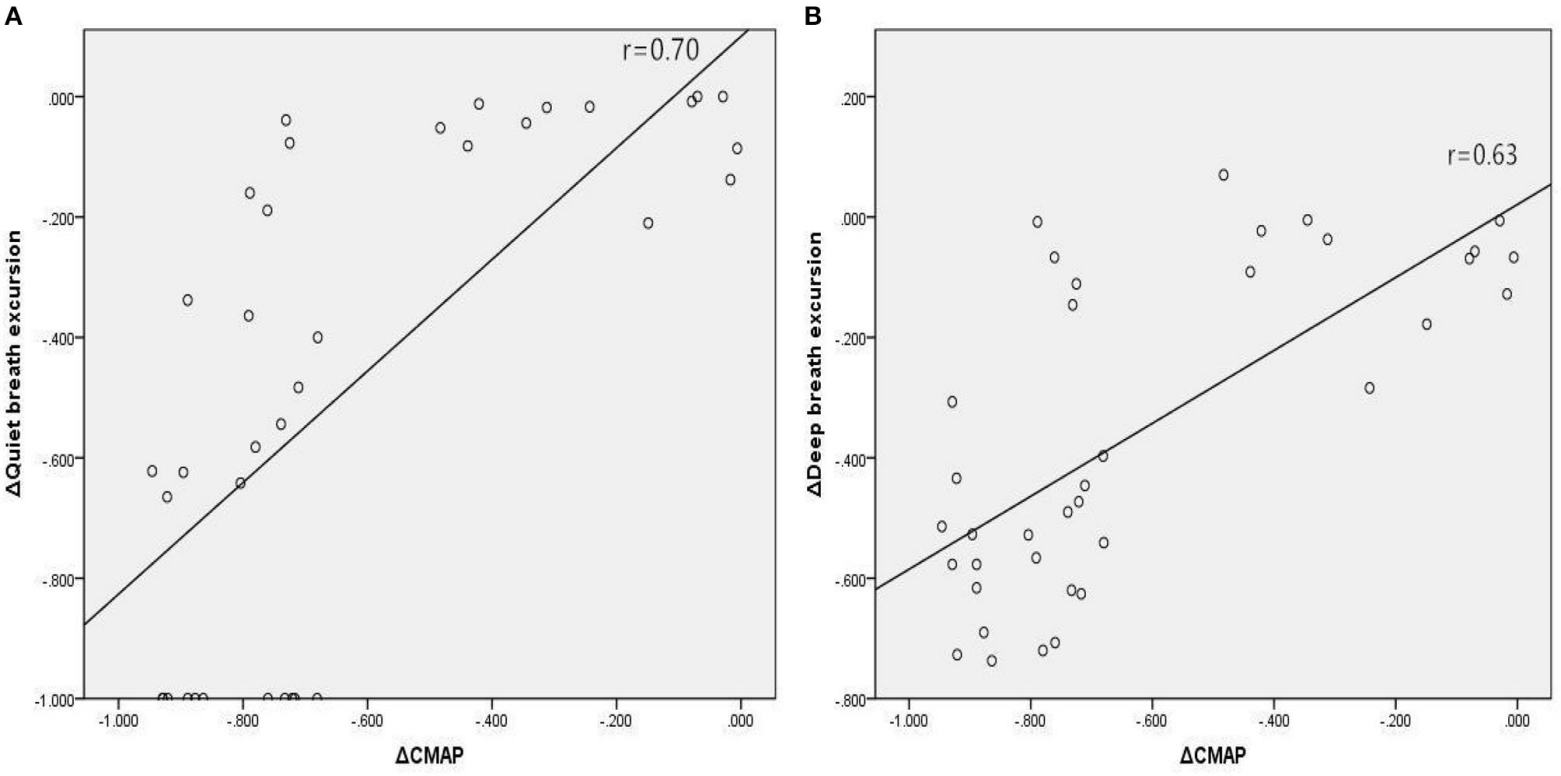
Associations between relative changes (Δ) in diaphragm CMAP amplitude and diaphragmatic excursions during quiet breathing **(A)** and deep breathing **(B)** before and 30 min after SCBPB in Group A. CMAP, compound muscle action potential.

**Figure 3:**
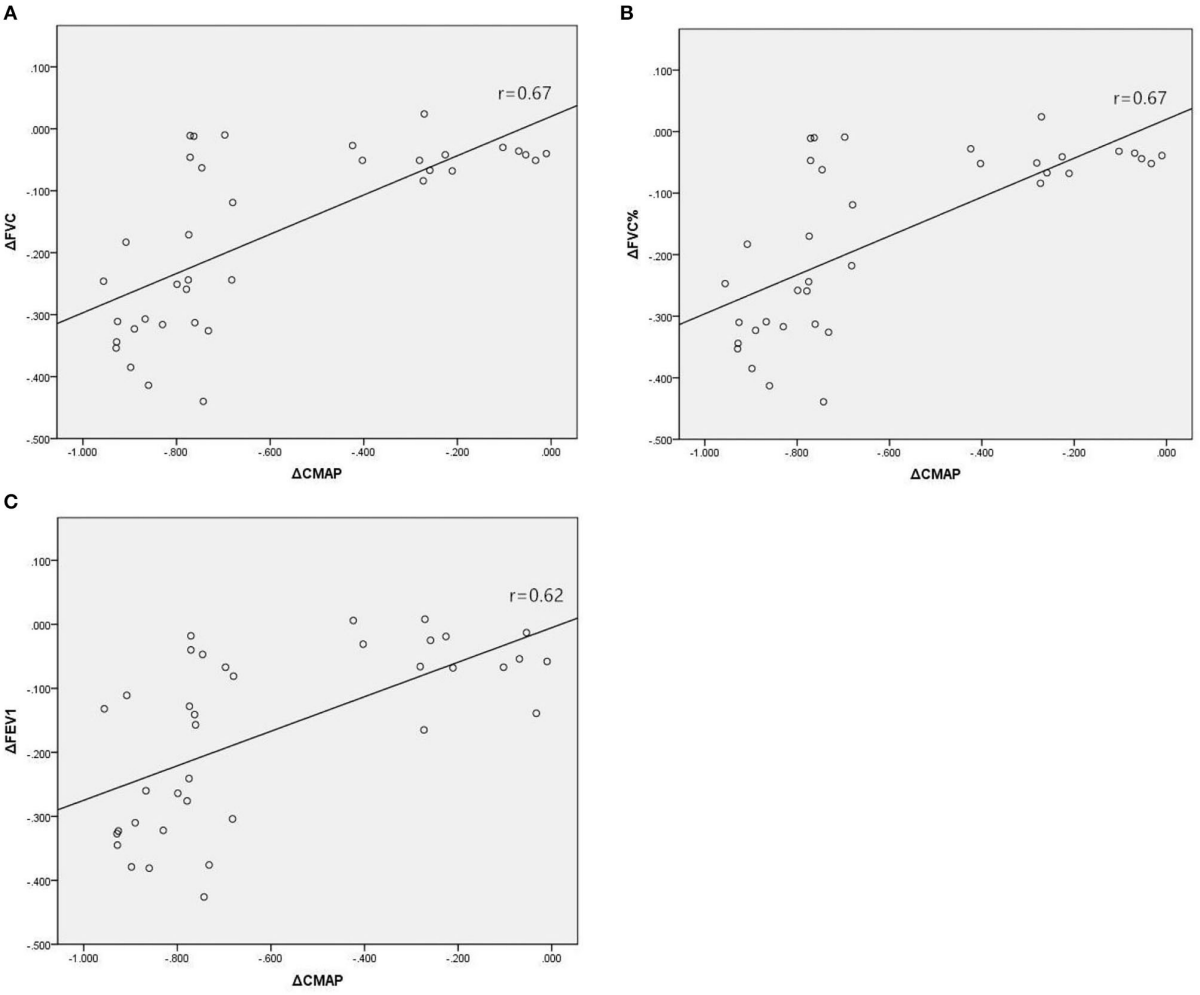
Associations between relative changes (Δ) in diaphragm CMAP amplitude and FVC **(A)**, FVC% **(B)**, and FEV1 **(C)** before and 30 min after SCBPB in Group B. CMAP, compound muscle action potential; FVC, forced vital capacity; FVC%, predicted value of FVC; FEV1, forced expiratory flow in the first second.

In Group A, there were significant associations between the reduction in amplitude of diaphragm CMAP and reductions in diaphragmatic excursion during quiet breathing (Figure 2A; *r* = 0.70, *p* < 0.001) and deep breathing (Figure 2B; *r* = 0.63, *p* < 0.001), when expressed as a percentage of baseline values. In Group B, there were significant associations between the reduction in amplitude of diaphragm CMAP and reductions in FVC (Figure 3A; *r* = 0.67, *p* < 0.001), FVC% (Figure 3B; *r* = 0.67, *p* < 0.001), and FEV1 (Figure 3C; *r* = 0.62, *p* < 0.001), when expressed as a percentage of baseline values.

### Diagnostic Efficacy of Pulmonary Function for Hemidiaphragmatic Paralysis

**Figure 4:**
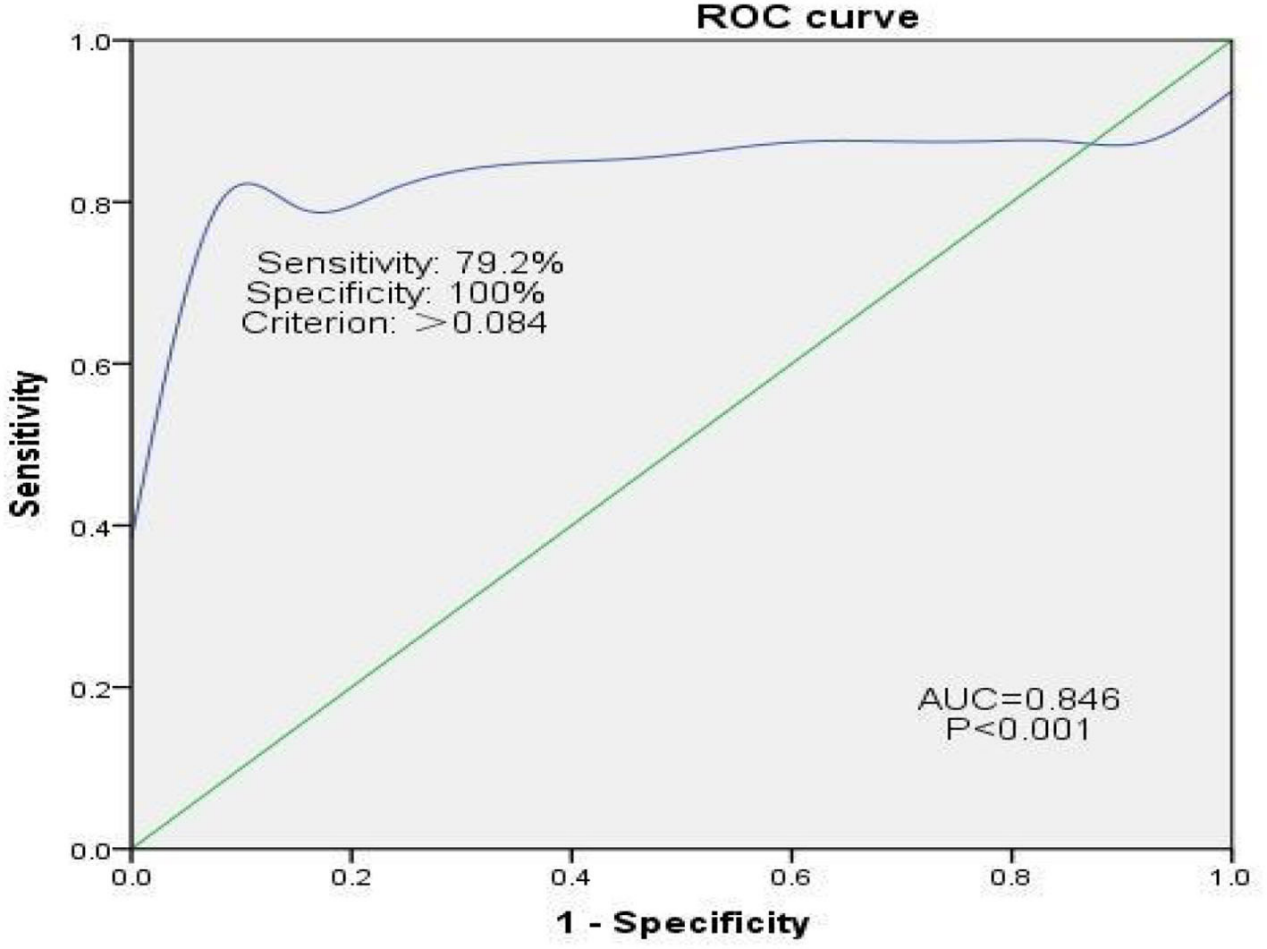
ROC curve analysis assessing the efficacy of pulmonary function for diagnosing hemidiaphragmatic paralysis evidenced by diaphragm CMAP as an assessment of diaphragm function. CMAP, compound muscle action potential. Sensitivity = True Positives/(True Positives + False Negatives). False Positive Rate = 1 – Specificity, where specificity = True Negatives/(True Negatives + False Positives).

In Group B, 24 of the 37 (64.9%) patients experienced hemidiaphragmatic paralysis. ROC curve analysis was used to assess the diagnostic efficacy of pulmonary function for hemidiaphragmatic paralysis evidenced by diaphragm CMAP as an assessment of diaphragm strength (Figure 4). The area under the ROC curve for FVC was 0.86. At a cut-off of 0.084, FVC predicted hemidiaphragmatic paralysis (determined by diaphragm CMAP) with sensitivity and specificity of 79.2 and 100%, respectively.

## Discussion

This study assessed the associations between diaphragm CMAP, hemidiaphragmatic excursion, and pulmonary function after SCBPB. The efficacy of pulmonary function for diagnosing hemidiaphragmatic paralysis evidenced by diaphragm CMAP as an assessment of diaphragm strength was evaluated. To date, the pulmonary function has not been used to indicate diaphragmatic paralysis. A decrease of >8.4% in FVC compared to pre-block has predicted hemidiaphragmatic paralysis (determined by diaphragm CMAP) with sensitivity and specificity of 79.2 and 100%, respectively.

The brachial plexus nerve is anatomically adjacent to the phrenic nerve and originates from the anterior rami of C5 ≈ 8 and T1. The phrenic nerve originates from the anterior rami of C3 to C5 and passes over the anterior surface of the anterior scalene. The accessory phrenic nerve originates from the anterior rami of C5 ≈ 6. The accessory phrenic nerve is a common anatomic variant that is present in up to 75% of individuals. Local anesthetic blockade of the accessory phrenic nerve may lead to diaphragmatic paralysis during brachial plexus block. Diaphragmatic paralysis may be a temporary or persistent phenomenon. The incidence of diaphragmatic paralysis after SCBPB in our patient population was high. Eight patients reported transient dyspnea and oxygen desaturation 10 min after SCBPB. These data highlight the unmet clinical need to raise awareness about diaphragmatic paralysis as a complication of brachial plexus block and identify novel methods to measure diaphragmatic paralysis. Previously, we explored the effect of 20 and 30 ml of 0.375% ropivacaine on electromyography of the diaphragm and pulmonary function before and after ultrasound-guided SCBPB (21).

In the present study, we showed a significant association between the reduction in amplitude of diaphragm CMAP after SCBPB, measured by PNCS, and the reductions in a diaphragmatic excursion on quiet and deep breathing, visualized on the ultrasound, when expressed as a percentage of baseline values. The association was better during quiet breathing compared to deep breathing. The diaphragm is responsible for 75% of tidal volume during quiet inspiration (22). This percentage is reduced during deep breathing due to the assistance of extrinsic musculature, which increases the volume of the thorax and expands the lungs (23).

In addition, there was a significant association between the reduction in amplitude of diaphragm CMAP after SCBPB and the reduction in pulmonary function (FVC, FVC%, and FEV1), when expressed as a percentage of baseline values. It is well-established that diaphragmatic paralysis is related to a restricted breathing pattern and reduced ability of the lung to expand, which is characterized by reduced FVC and FVC% (24). There was a moderate association between the pre-block and post-block changes in diaphragm CMAP and FVC, FVC%, and FEV1. During unilateral diaphragmatic paralysis, the contralateral diaphragm may compensate for the dysfunction of the paralyzed hemidiaphragm, and patients can maintain normal ventilation at rest and during mild exercise. Patients may not have signs or symptoms until FVC falls to 38% of predicted due to physiological compensation (25, 26).

Results of our ROC curve analysis imply that hemidiaphragmatic paralysis after SCBPB, evidenced by diaphragm CMAP as an assessment of diaphragm strength, may be diagnosed when FVC falls below 0.084. The area under the ROC curve was 0.85, and sensitivity and specificity were 79.2 and 100%, respectively. Consistent with this, Ming-Lung et al. reported that phrenic nerve transfer for the repair of avulsed brachial plexus injury resulted in permanent ipsilateral diaphragmatic paralysis accompanied by an estimated 8% decrease in inspiratory capacity, FVC, and total lung capacity (27).

Several methods have been used to diagnose hemidiaphragmatic paresis after the brachial blockade, such as fluoroscopy, maximum transdiaphragmatic pressure, PNCS CMAP amplitude, and ultrasound of the diaphragm. Each method has its advantages and disadvantages. Ultrasound of the diaphragm has advantages over diaphragm CMAP as it is easy to apply. However, ultrasound does not account for the influence of the contralateral diaphragm and accessory respiratory muscles. PNCS can overcome these deficiencies. In this study, we used needles instead of surface electrodes to collect diaphragm CMAP. As surface electrodes are distant from the deep target muscle, the signal can be influenced by factors, such as lung inflation, skin thickness, the amount of subcutaneous tissue, and the depth of lung tissue separating the recording electrode from the diaphragm. Needles are not prone to interference from activity in neighboring muscles and are considered safe (17); however, this approach can be technically challenging. Inaccurate placement of needles can make it difficult to collect diaphragmatic needle electromyographic recordings, providing inaccurate measurements of the latency and amplitude of the diaphragm CMAP.

Pulmonary function is widely used to evaluate the effect of brachial plexus block on respiratory function. It is simple and easy to perform, but the quality of the spirometry test is influenced by patient efforts and cooperation, and there are no diagnostic criteria for diaphragmatic paralysis. Diaphragm CMAP has been applied in the diagnosis of respiratory insufficiency due to amyotrophic lateralizing sclerosis and other neuromuscular disorders (28), but data describing the use of diaphragm CMAP to diagnose diaphragmatic paralysis after SCBPB are limited. The majority of previous reports have used ultrasound to diagnose hemidiaphragmatic paralysis after SCBPB. With the rapid development and decreasing cost of ultrasound technology, real-time 2-dimensional visualization of the diaphragm can be easily performed at the bedside (29). However, ultrasound may misdiagnose complete paralysis of the phrenic nerve as diaphragmatic movement on the ipsilateral side can be affected by movement from the contralateral diaphragm and accessory respiratory muscles (30). PNCS of the affected hemidiaphragm is needed to confirm complete paralysis of the phrenic nerve. The phrenic nerve is the only source of motor innervation to the diaphragm (24). Therefore, the electrophysiological function of the diaphragm can be analyzed without being affected by other respiratory muscles.

This study was associated with several limitations. First, the latency and amplitude of the diaphragm CMAP were extremely atypical in several patients. This may have been due to interference caused by stimulation of the brachial plexus. Second, patients with a high BMI or cardiopulmonary disease were excluded from this study. This may limit the generalizability of our findings, as these patients are most likely to be affected by diaphragmatic paralysis. Third, the left hemidiaphragm is lower than the right hemidiaphragm, but the motion of the left hemidiaphragm is greater than the right hemidiaphragm (24); therefore, measurements from the right hemidiaphragm cannot be extrapolated to the left. Fourth, it takes 2 min to measure diaphragmatic excursions and 3 min to measure pulmonary function. As we could not adjust for the differences in the time it takes to obtain these measurements, we did not explore the relationship between diaphragmatic excursion and pulmonary functions. Although diaphragm dysfunction (CMAP) was identified by two different approaches [ultrasound and pulmonary function tests (PFTs)] in two groups of patients, the characteristics of the patients in Groups A and B were similar, allowing for a relevant extrapolation of data.

## Conclusions

Diaphragm CMAP, determined by PNCS, diaphragmatic excursion during quiet and deep breathing, and pulmonary function (FVC, FVC%, and FEV1) were decreased after SCBPB, implying these three methods can be used to diagnose diaphragmatic paralysis. The pulmonary function can directly reflect the influence of diaphragmatic paralysis on respiratory function after SCBPB. A decrease of >8.4% in FVC compared to pre-block has predicted hemidiaphragmatic paralysis (determined by diaphragm CMAP). The relative reduction in diaphragm CMAP amplitude was correlated well with the relative reductions in diaphragmatic excursion and pulmonary functions. These data establish diaphragm CMAP as an accurate and objective index of diaphragmatic paralysis.

## Data Availability Statement

The original contributions presented in the study are included in the article/Supplementary Material, further inquiries can be directed to the corresponding author/s.

## Ethics Statement

The studies involving human participants were reviewed and approved by Ethical Committee of 98th Hospital of PLA. The patients/participants provided their written informed consent to participate in this study.

## Author Contributions

XB is responsible for conducting this study and the manuscript writing. Data collection and analysis were completed by TL and YZ. YW is responsible for implementation of clinical trials. This study was designed by XK. After completing this clinical study and article writing, XW was responsible for the revision of the manuscript. All authors contributed to the article and approved the submitted version.

## Funding

This work was supported by Bethune Charitable Foundation (BCF-RF-WSQZTZJ-202011-031) and the Public Welfare Research Projects of Huzhou Technology Division (2018 GYB41).

## Conflict of Interest

The authors declare that the research was conducted in the absence of any commercial or financial relationships that could be construed as a potential conflict of interest.

## Publisher's Note

All claims expressed in this article are solely those of the authors and do not necessarily represent those of their affiliated organizations, or those of the publisher, the editors and the reviewers. Any product that may be evaluated in this article, or claim that may be made by its manufacturer, is not guaranteed or endorsed by the publisher.
